# Breast cancer recurrence after sentinel lymph node biopsy

**DOI:** 10.12669/pjms.316.8427

**Published:** 2015

**Authors:** Abdulaziz AlSaif

**Affiliations:** 1 Dr. Abdulaziz A. Alsaif, Associate Professor of Surgery, King Saud University, Department of Surgery, P.O. Box 59854, Riyadh 11535, Saudi Arabia

**Keywords:** Breast cancer, Cancer recurrence, Sentinel lymph node biopsy

## Abstract

**Objective:**

To look into the pattern of breast cancer recurrence following mastectomy, breast conservative surgery and radiotherapy or chemotherapy after SLNB at our institution.

**Methods:**

Between January 2005 and December 2014, all patients diagnosed with breast cancer with clinically negative axilla, underwent SLNB. We reviewed their medical records to identify pattern of cancer recurrence.

**Results:**

The median follow-up was 35.5 months. Eighty five patients (70.8%) had a negative sentinel lymph node (SLN) and subsequently had no further axillary treatment, one of them (1.2%) developed axillary recurrence 25 months postoperatively. Twenty five patients (20.8%) had a positive SLN (macrometastases) and subsequently had immediate axillary lymph node dissection (ALND). Ten patients (8.3%) had a positive SLN (micrometastases). In the positive SLN patients (macrometastases and micrometastases), there were two ipsilateral breast recurrences (5.7%), seen three and four years postoperatively. Also in this group, there was one (2.9%) distant metastasis to bone three years postoperatively.

**Conclusion:**

In this series, the clinical axillary false negative rate for SLNB was 1.2% which is in accordance with the published literature. This supports the use of SLNB as the sole axillary staging procedure in breast cancer patients with negative SLNB. Axillary lymph node dissection can be safely omitted in patients with micrometastases in their sentinel lymph node(s).

## INTRODUCTION

Axillary lymph node (ALN) status is the most powerful prognostic factor in patients with breast cancer and it determines, along with the biological characteristics of the primary tumor, the prognosis and the subsequent adjuvant treatment.^1^,^2^ Axillary lymph node dissection (ALND) has long been considered as the standard surgical treatment for the axilla in breast cancer.^3^,^4^

The more frequent use of mammograms and the increased public awareness of breast cancer have led to a decrease in tumor size at presentation, where the chances of axillary lymph nodes metastases is about 40%.^5^-^7^ This 40% may benefit from ALND, however, the remaining 60% will not benefit from ALND, rather, they may suffer from the ALND-associated morbidities.^8^-^10^

Sentinel lymph node biopsy (SLNB), a minimally invasive procedure which was introduced in the early 1990’s, has become the standard of care for staging the clinically negative axilla in breast cancer patients as it accurately reflects the status of the remaining axillary lymph nodes and has an excellent identification rate.^11^-^13^

Studies in the literature have shown very low rates of locoregional recurrence after omitting ALND in sentinel node negative patients.^14^ The results of recent trials showed that ALND can be safely omitted in early breast cancer patients with micrometastases in their sentinel nodes (SN).^15^ The aim of this study was to look in to the pattern of cancer recurrence after SLNB procedure at our centre.

## METHODS

**Fig.1:**
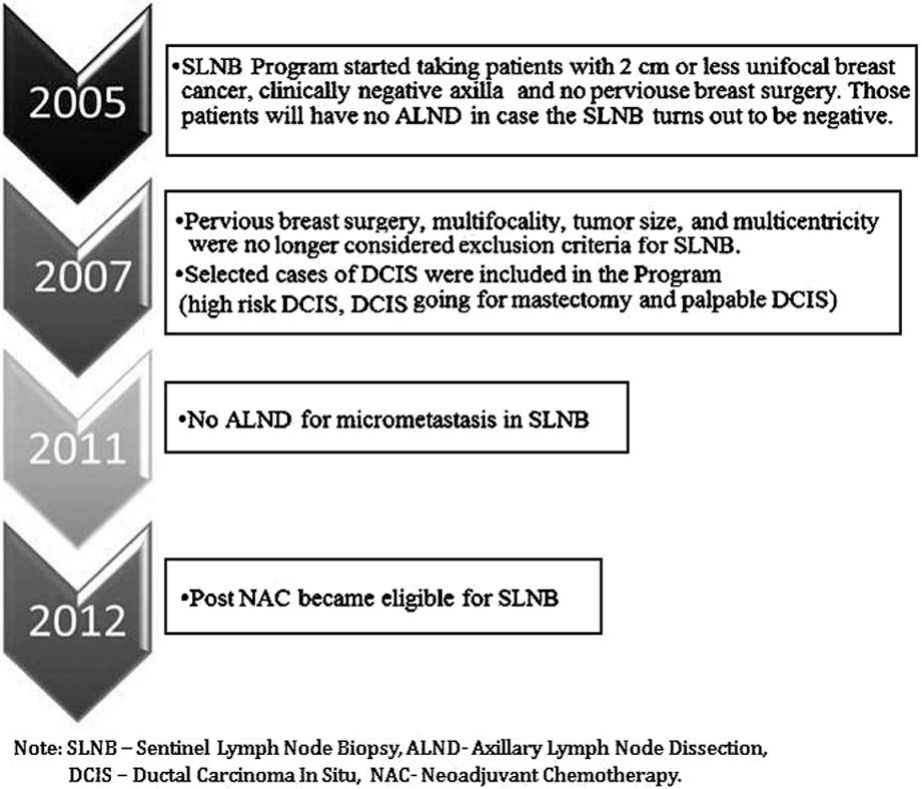
Beginning and development of SLNB program.

Between January 2005 and December 2014, a total of 120 female patients diagnosed to have breast cancer with clinically negative axilla, had SLNB at our institution. Three patients had bilateral disease. The sentinel node could not be identified in three patients, so ALND was carried out immediately on them. Beginning and development of the SLNB program at our centre is shown in Fig.1. The SLNB program at our centre was started by one of our surgeons who had a formal overseas training on this procedure where he attained the initial learning phase and validation of the procedure. We started our program in 2005 by taking patients with tumor size of 2.0 cm or less and who fulfill the other criteria (Fig.1), but as time went by, we modified our inclusion criteria based on evolving scientific evidence at time of modification. All decisions concerning the patient’s treatment plan were discussed in a multidisciplinary setting.

### Lymphatic mapping and operative procedure

Sentinel lymph node mapping is done by using the dual method (radiolabeled colloid and a vital blue dye). Tc 99m sodium pertechnetate (1.5ml) with activity ranging from 5 – 100 mCi (185 – 3700 MBq) is introduced in a vial. Four syringes are prepared from this vial, each syringe containing 0.1 – 0.2 ml Tc99m albumin colloid containing 0.2 – 0.4 mCi (7.4 – 14.4 MBq). This material is injected at four peritumoral sites or at the periareolar area in case of multicentric cancer. Gentle breast massage is applied to facilitate passage of the particles towards the axilla. Post injection imaging is done using Gamma camera (Phillips SPECT-CT system). Two and a half ml of sterile saline plus 2.5 ml of the blue dye (Bleu Patente V, Sodique Guerbet 2.5%, France) are mixed and injected subdermally peritumorally just after induction of anesthesia, the breast is massaged for 5 minutes. The axilla is explored 10 – 15 minutes after blue dye injection. A lymph node is called SN when it is blue stained or there is a blue lymphatic channel leading to it, or when it is a hot node by using the gamma detector probe (GPS Navigator GPS-9100-00 Dynasil, USA). After removal of the SN, the axilla is checked for residual radioactivity, a count less than 10% of the hottest sentinel node is considered a background activity.

### Pathologic Examination of the SLNB

All sentinel nodes (SN) were sent for frozen section examination. Each SN is sliced perpendicular to its long axis, touch preparation are submitted from the cut surfaces and stained with modified Giemsa stain (Diff Quick). The entire node(s) is /are submitted after slicing for frozen section. The slices are performed in an equidistant manner of 2mm thickness for each slice. Immunohistochemistry (IHC) is used for doubtfully negative hematoxylin and eosin (H&E) results. Micrometastases are defined based on a size greater than 0.2 mm and less than or equal to 2 mm in diameter according to the AJCC classification.^15^ Isolated tumor cells or tumor cell clusters measuring less than 0.2 mm in diameter did not meet the definition of micrometastases, and were considered node negative. Patients with SN macrometastases had an immediate ALND (level I and level 2). Patients with negative SN had no further axillary treatment. In this study, there were 10 patients with SN micrometastases, six of them had delayed ALND (early phase of study), the other four had no ALND (late phase of study).

### Adjuvant therapy

Seventy one patients (59.2%) had breast – conserving therapy (BCT). The rest of the patients had mastectomy. These patients received post-operative radiation therapy with 45 Gy over five weeks with a boost dose of 10 Gy to the tumor bed which was marked with surgical clips during surgery. No radiation was given to the axilla. Hormonal therapy (Tamoxifen), 20 mg daily for five years was given to 85 patients (69.1%). Biological therapy was given to 25 patients (20.3%), 17 cycles of Trastuzumab, one cycle every three weeks over one year period of time. Chemotherapy was given according to the protocol at our Oncology Unit.

### Patients follow-up

All patients were clinically examined every three months in the first postoperative year, then every six months in the years after (examination of the breasts or chest wall, axillae, supraclavicular fossae). Annual mammogram is done. Dedicated breast ultrasound is done if needed. Other hematological and imaging investigations are done as the clinical situation dictates. We lost follow up of 14 patients out of 117 (11.9%)

### Statistical analysis

All data collected were encoded into a Microsoft Excel program and analyzed using the Predictive Analysis for Social Sciences (PASW) software version 19.0 (SPSS, IBM, Chicago, Illinois, USA). Categorical results are reported as frequencies (n) and percentages (%).

## RESULTS

The patient’s demographics, tumor characteristics and the primary breast surgical procedure for our study population are shown in Table-I. The median patients’ age was 48 years. Seventy eight of our patients (65.0%) were premenopausal. One hundred six patients (86.2%) had invasive ductal carcinoma. The median pathologic tumor size was 2.7cm (range: 1.5 - 6.9 cm). Seventy nine patients (64.2%) had T2 tumors. Grade 2 tumors were seen in 82 patients (66.7%). Lymphovascular invasion and perineural invasion were seen in 26 (21.1%) and 15 (12.2%) patients, respectively. Breast – conserving therapy was done to 71 patients (59.2%).

**Table-I:** Patients demographics, tumor characteristics and primary breast surgical procedure (120 patients, 3 patients had bilateral cancer).

Characteristics	n (%)
Age in years, mean (standard deviation)	49.2 (10.4)
Age in years, median	48.0
*Menopausal status*	
• Pre- menopausal	78(65.0)
• Post- menopausal	42(35.0)
*Type of tumor*	
• Ductal	106 (86.2)
• Lobular	11(8.9)
• DCIS	6(4.9)
Pathologic T Stage (median pathologic tumor size, cm (range)	2.7 (1.5-6.9)
• T1	40 (32.5)
• T2	79 (64.2)
• T3	4 (3.3)
*Tumor grade*	
• I	11 (8.9)
• II	82 (66.7)
• III	30 (24.4)
Lymphovascular invasion	26 (21.1)
Perineural invasion	15 (12.2)
*Location of Tumor*	
• Upper outer quadrant	65 (52.9)
• Upper inner quadrant	16 (13.0)
• Lower outer quadrant	14 (11.4)
• Lower inner quadrant	11 (8.9)
• Central	17 (13.8)
*Affected breast side*	
• Right side	66 (55.0)
• Left side	51 (42.5)
• Bilateral	3 (2.5)
With multifocal disease	20 (16.7)
With multicentric disease	10 (8.3)
Received neoadjuvant chemotherapy before SLNB	18 (15.0)
Estrogen receptor positive	85 (69.1)
Progesterone receptor positive	76 (61.8)
Her2 receptor positive	25 (20.3)
*Primary Breast Surgical procedure*	
• Mastectomy	49(40.8%)
• Breast-conserving therapy (BCT)	71(59.2%)

Table-II show that in 115 patients (95.8%), the number of retrieved SN was between 1 – 3 nodes. The number of positive SN was one node in 21 patients (60%), two nodes in 11 patients (31.4%), and three nodes in three patients (8.6%). Table-III show that there were 123 mapping procedures, in three of them, the SN could not be identified so they had immediate ALND. Our identification rate was 97.6%. Eighty five patients (70.8%) were SN negative and had no further axillary treatment. Twenty five patients (20.8%) were found to have a positive SN (macrometastases) and underwent immediate ALND. Ten patients (8.3%) were later found to have micrometastases by IHC, six of those ten patients had delayed ALND (early phase of study) while the remaining four had no further axillary treatment. Sentinel node(s) was/were the only positive node(s) in the axillary basin in 23 ALND specimens out of 31 (74.2%).

**Table-II:** Sentinel lymph node results.

*Number of Sentinel Lymph Nodes harvested*	
• One lymph node	36(30.0%)
• Two lymph nodes	60(50.0%)
• Three lymph nodes	19(15.8%)
• Four lymph nodes	3(2.5%)
• Five lymph nodes	2(1.7%)
*Number of Positive Sentinel Lymph Nodes*
• One node	21 patients (60.0%)
• Two nodes	11 patients (31.4%)
• Three nodes	03 patients (08.6%)

**Table-III:** Sentinel lymph node (SLN) histopathology.

Number of sentinel node mapping	123
Number of sentinel node not found	03
Identification rate	97.6%
Number of SLN negative	85/120 (70.8%)
Number of SLN positive	35/120 (29.2%)
Number of SLN macrometastases	25/120 (20.8%)
Number of SLN micrometastases	10/120 (8.3%)
SLN positive, ALND positive	8/31 (25.8%)
SLN positive, ALND negative	23/31 (74.2%)

The median patient follow up was 35.5 months and the mean was 38.8 months. In this study population, the recurrence – free survival was 100% in the first 2 years. In the SN negative patients, there was one event (1.2%) which occurred 25 months after doing SLNB for a 41-year old patient. The recurrence was a clinically palpable axillary mass. The SN was negative by H&E and IHC, the patient’s choice was for mastectomy, so no radiotherapy was given. This patient’s index tumor was 3.5cm in size, grade 3, lymhovascular invasion was present, Estrogen and Progesterone receptors negative, HER-2/neu receptor positive, this patient received chemotherapy and Biological therapy. The recurrence was dealt with by doing ALND, which retrieved 18 lymph nodes, one of them was grossly metastatic.

In the SN positive patients, three patients (8.6%) out of 35 (macrometastases and micrometastases) developed a recurrence. There was one bone recurrence (2.9%), and two ipsilateral breast recurrences (5.7%). None of the patients who had micrometastases in their SN developed a recurrence. Table-IV shows the site and frequency of cancer recurrence in both SN negative and SN positive patients. There were two deaths in this series, one was breast cancer related (T3, triple negative, 35 years old, SN positive patient). The other death was due to a cardiac comorbidity.

**Table-IV:** Site and frequency of cancer recurrences in SN negative and SN positive patients.

Site of recurrence	SN negative patients (n=85)	SN positive patients (n=25)	SN micromtastases Patients (n=10)
Ipsilateral Axilla	01 (1.2%)	0	0
Ipsilateral Breast (post BCT)	0	02(5.7%)	0
Distant	0	01(2.9%)	0
Total	01(1.2%)	03(8.6%)	0

SN = Sentinel Node, BCT = Breast conserving therapy.

## DISCUSSION

Sentinel lymph node biopsy is the standard of care for staging the clinically negative axilla in breast cancer patients.^11^-^13^ In a meta- analysis of SLNB procedure in breast cancer which included 912 patients in 11 studies, a false negative rate of 5% was reported for this procedure,^16^ yet a significantly fewer clinically overt axillary recurrences are seen post negative SLNB than would be expected based on the reported false negative rate of SLNB procedure. Clinical axillary recurrence rate after a negative SLNB ranges between 0 - 1.5% at a median follow up of 46 and 26 months respectively.^17^,^18^ In our study, 1.2% of SN negative patients developed disease recurrence during a median follow- up period of 35.5 months; this single axillary failure was seen 25 months post a negative SLNB in a young patient with unfavorable tumor biological characteristics.

Many factors may explain the discrepancy between the false negative rate of SLNB and the overt clinical axillary failure post negative SLNB; the more liberal use of systemic adjuvant therapy which sterilizes and downstages positive lymph nodes,^19^ the rather short median follow – up period of some published studies,^20^,^21^ the possibility of incorporating the lower axillary region into the radiation field directed at the breast, and probably the ever – improving sentinel lymph node identification rate in recent studies.^22^

It is worth mentioning that the clinical axillary failure after a complete ALND ranges between 0% - 1.4%.^11^,^23^ In this study, 8.6% of the SN positive patients, who had subsequent ALND, developed disease recurrence outside the axilla. Despite having received breast irradiation, there were 2 local recurrences (5.7%) which were seen 3 & 4 years post SLNB procedure respectively, which is in line with published literature,^24^ also in the SN positive patients, there was a single systemic failure (2.9%) which was seen 3 years post SLNB procedure.

Two randomized phase 3 trials have been conducted to look into whether ALND can be safely omitted in early breast cancer patients with positive SN. In the American College of Surgeons Oncology Group Z0011 trial (ACOSOG Z0011 trial), clinically node-negative early breast cancer patients with positive SN were randomized to ALND or no ALND groups. At a median follow-up time of 6.3 years, there were no statistically significant differences in local recurrence or regional recurrence between the two2 groups.^24^ The International Breast Cancer Study Group trial (IBCSG 23-01 trial) randomized patients with micromestatases or isolated tumor cells (ITCs) in the SN to ALND versus no ALND, after a median follow-up time of 5 years, the results showed no significant difference in five-year disease-free survival or locoregional recurrence rates between the two groups.^25^

In our study, 28.6% (10/35) of all SN metastases were micrometastases, six of them had delayed ALND (early years of the study) which revealed negative axillary lymph nodes, the other four (later years of the study) had no further axillary treatment. After a median follow – up time of 35.5 months, no local, regional, or distant recurrence were seen in this subset of the study population.

In conclusion, the low incidence of clinical axillary failure in SN negative patients supports the use of SLNB as the sole axillary staging procedure in breast cancer patients with negative SLNB. ALND can be safely omitted in patients with micrometastases in their sentinel lymph node(s).
